# Securing web applications against XSS and SQLi attacks using a novel deep learning approach

**DOI:** 10.1038/s41598-023-48845-4

**Published:** 2024-01-20

**Authors:** Jaydeep R. Tadhani, Vipul Vekariya, Vishal Sorathiya, Samah Alshathri, Walid El-Shafai

**Affiliations:** 1 Gujarat Technological University, Ahmedabad, Gujarat India; 2 Parul Institute of Engineering and Technology, Parul University, Vadodara, Gujarat India; 3 Department of Information Technology, College of Computer and Information Sciences, Princess Nourah bint Abdulrahman University, P.O. Box 84428, 11671 Riyadh, Saudi Arabia; 4 Security Engineering Lab, Computer Science Department, Prince Sultan University, 11586 Riyadh, Saudi Arabia; 5 Department of Electronics and Electrical Communications Engineering, Faculty of Electronic Engineering, Menoufia University, Menouf, 32952 Egypt

**Keywords:** Computer science, Engineering

## Abstract

Modern web application development involves handling enormous amounts of sensitive and consequential data. Security is, therefore, a crucial component of developing web applications. A web application's security is concerned with safeguarding the data it processes. The web application framework must have safeguards to stop and find application vulnerabilities. Among all web application attacks, SQL injection and XSS attacks are common, which may lead to severe damage to Web application data or web functionalities. Currently, there are many solutions provided by various study for SQLi and XSS attack detection, but most of the work shown have used either SQL/XSS payload-based detection or HTTP request-based detection. Few solutions available can detect SQLi and XSS attacks, but these methods provide very high false positive rates, and the accuracy of these models can further be improved. We proposed a novel approach for securing web applications from both cross-site scripting attacks and SQL injection attacks using decoding and standardization of SQL and XSS payloads and HTTP requests and trained our model using hybrid deep learning networks in this paper. The proposed hybrid DL model combines the strengths of CNNs in extracting features from input data and LSTMs in capturing temporal dependencies in sequential data. The soundness of our approach lies in the use of deep learning techniques that can identify subtle patterns in the data that traditional machine learning-based methods might miss. We have created a testbed dataset of Normal and SQLi/XSS HTTP requests and evaluated the performance of our model on this dataset. We have also trained and evaluated the proposed model on the Benchmark dataset HTTP CSIC 2010 and another SQL/XSS payload dataset. The experimental findings show that our proposed approach effectively identifies these attacks with high accuracy and a low percentage of false positives. Additionally, our model performed better than traditional machine learning-based methods. This soundness approach can be applied to various network security applications such as intrusion detection systems and web application firewalls. Using our model, we achieved an accuracy of 99.84%, 99.23% and 99.77% on the SQL-XSS Payload dataset, Testbed dataset and HTTP CSIC 2010 dataset, respectively.

## Introduction

Modern web-based and cloud-based applications have become the primary way individuals access digital services. However, they also present a significant security concern. Vulnerabilities in coding, weaknesses, and the leakage of sensitive data can all be exploited by cybercriminals. A report by McAfee in 2021^1^ estimated the cost of cybercrime to be over $1400 billion in 2020. In another report by SonicWall, they showed ransomware attacks, Phishing attacks, File-less attacks and Encrypted malware attacks happened 91%, 76%, 39%, and 66%, respectively^2^. Therefore, security experts must create tools that detect and prevent such attacks. They also design new web-based structures that decrease the opportunity for web-based attacks^3^. Eavesdropping^4^ and poisoning attack^5^ are also very much severe which may cause confidential information leakage or inaccessible data.

The rapid development of internet usage led to a significant increase in web applications, which are crucial for companies to offer their services. These applications heavily rely on databases that store and transmit the requested data to the users. These databases are often targeted with attacks^6^, the most common being cross-site scripting (XSS) SQL injection^7^. SQLi attacks exploit database security vulnerabilities by injecting malicious code into database queries, granting access to data and allowing modification of it.

XSS attacks function similarly, but the malicious JavaScript code is inserted into web applications and websites, redirecting users to malicious websites. By analyzing massive amounts of data and finding patterns that may point to an attack, deep learning is a potent machine learning approach used to identify web application threats^8^. In intrusion detection systems^9^, deep learning is frequently used to detect web application threats (IDS). These systems use deep learning algorithms to Examine network traffic for odd patterns or abnormalities that might point to an attack. Another use case is in web application firewalls (WAF)^10^, which uses deep learning algorithms to analyze and classify web traffic to identify and block malicious requests. Deep learning techniques have also been applied to detect cross-site scripting attacks SQL injection by analyzing server logs and identifying patterns that may indicate an attack.

### Various web application attacks

- SQL injection: by inserting malicious SQL code into a web application, an attacker can access a database without authorization and potentially steal sensitive data^11^.
- XSS Attack: in this attack, by injecting malicious code into a web page to be viewed by other users, this malicious code can then be executed by the browser, potentially stealing sensitive user information or performing other malicious actions^12^.
- Cross-site request forgery: including changing a password or making a purchase, Inadvertent activities on a website might be carried out by a user due to this kind of attack^13^.
- File inclusion vulnerabilities: two file inclusion vulnerabilities exist: local file inclusion (LFI) and remote file inclusion (RFI). Unlike RFI, which enables attackers to include and execute distant files, LFI only permits access to local files on the server^14^.
- Distributed denial of service (DDoS): by overwhelming a website with traffic from numerous sources, this assault prevents legitimate people from accessing it^14^.
- Unvalidated inputs: this vulnerability allows an attacker to input arbitrary data into a web application, potentially allowing them to bypass security controls or gain unauthorized access.
- Malicious file execution: it allows an attacker to upload a malicious file to a website, which can then be executed by the server, potentially giving the attacker access to sensitive information.
- Cookie poisoning: this attack occurs when an attacker modifies a cookie, potentially allowing them to gain unauthorized access to a website^15^.
- Weak session IDs: in this vulnerability, the attacker can use various methods and techniques to predict or guess a user's session ID, potentially allowing them to take over that user's session^15^.
- Clickjacking: this attack tricks a user into clicking on a link or button, which can perform an action they did not intend, such as making a purchase or transferring funds^15^.
- Phishing: this attack uses social engineering techniques to trick users into providing personal information or login credentials^16^.
- Insecure cryptographic storage: this type of vulnerability occurs when sensitive information is not encrypted correctly, potentially allowing an attacker to access it^17^.
- Insufficient transport layer protection: this type of vulnerability occurs when data is transmitted over an insecure network, potentially allowing an attacker to intercept and view sensitive information^18^.

### SQL injection

A type of cyberattack known as SQL Injection attacks web applications by inserting malicious database code into a website's input fields to obtain access to the back-end database. SQL Injection can be used for stealing sensitive data such as user IDs or passwords, personal information, and financial information. Different kinds of SQLi Attacks^7^, including:

- Union-based SQL injection: in this technique, the UNION operator combines the output of multiple SQL statements to access sensitive data.
- Boolean-based SQL injection: this method uses true or false statements to determine the database schema and gain access to sensitive data.
- Time-based SQL injection: this kind of SQLi method delays the execution of SQL statements to extract information from the database.
- Stacked queries SQL injection: this method uses multiple SQL statements separated by semicolons to extract data from the database.
- Blind SQL injection: this method does not rely on error messages. Instead, it uses the time delay in the web page's response to extract sensitive information from a database.
- Error-based SQL injection: this method is based on error messages generated by the database to extract information about the database structure and sensitive data.

To prevent SQL Injection attacks^7,11^, prepared statements, parameterized queries, and validated user input-like methods are used by developers. It is also necessary to secure the database by implementing proper authentication, access control, and encryption. The primary cause of SQLIAs and other security risks is developers' lack of prior consideration of structured security techniques and a flexible, workable policy framework for mitigating risks. Furthermore, when such methods are considered, attackers try to create new strategies that can get around the defenses created by developers; they start using various methods to carry out the SQLIA.

### Challenges in SQLi attack detection

- Effective input validation can be challenging because attackers can circumvent filters by encoding payloads, exploiting comments, and utilizing other obfuscation techniques. It might be challenging to create and maintain thorough input validation rules^19^.
- Compared to regular SQL injection attacks, blind SQL injection attacks are more challenging to identify and counter. In a blind SQL injection attack, the attacker intends to damage web data.
- Many web-based applications are constructed on outdated codebases that might not have been security-conscious when they were first created. Finding and resolving SQL injection vulnerabilities in these systems can be difficult and time-consuming.

### XSS attack

**Figure 1:**
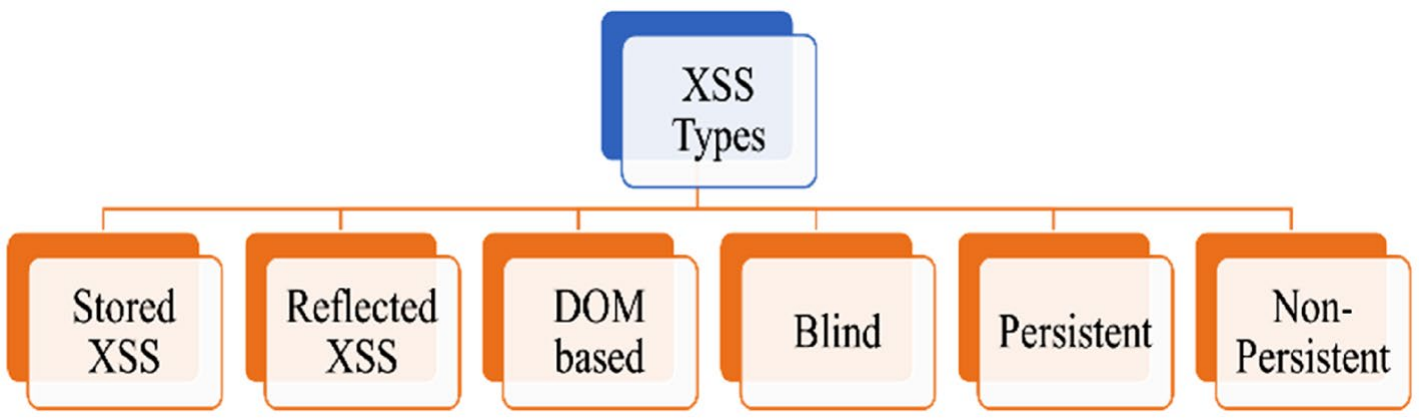
Different types of cross-site scripting attacks.

A cross-site scripting attack^20^ is a web application vulnerability that allows attackers to inject malicious code into a web page that other users can view. This attack can steal sensitive info like session IDs and cookies, redirect users onto malicious sites, or perform other malicious actions. There are several types of XSS attacks, as depicted in Fig. 1 below, which include:

- Stored XSS: when the malicious code is kept on the server and run each time a user accesses the compromised web page, an attack of this type occurs.
- Reflected XSS: in this attack, the attacker injects malicious code via a URL parameter reflected to the user.
- DOM-based XSS: a web page's document object model (DOM) is the target of this attack, which involves injecting malicious script for the browser to run.
- Blind XSS: this type of attack occurs when the attacker cannot see the results of the injected code, but the victim's browser still executes the injected code.
- Persistent attacks of XSS: this assault interacts with web pages instead of the non-persistent attack, representing a result. This attack also employs an injection script that unavoidably impacts the server's databases in various ways, including comment areas, logs, forums, etc. The victim then requests the previously saved information, and probably contains an injected script.
- Non-persistent attacks of XSS: the technique described is a reflective attack because when a user requests a service, the web server responds by reflecting its response. This service might be anything from mirrored messages to search results or any other kind of response that incorporates information sent to the server.

#### Challenges in XSS attack detection

- XSS assaults can be reflected, stored, or DOM-based, among other types. Multifaceted protection solutions are needed to defend against these attack vectors since various countermeasures may be needed for each type.
- Understanding the context in which user inputs are used is essential to accurately identifying and combating cross-site scripting (XSS) attacks. This context can change even within the same application, making it challenging for an automated system to identify^21^.
- Multiple origin material on a web page makes XSS assaults more likely to be complicated. Safeguarding security in these situations, particularly when utilizing Cross-Origin Resource Sharing (CORS), necessitates meticulous configuration to thwart assaults while permitting lawful cross-origin queries.

As discussed, many different tactics are used in SQL injection and XSS attacks. We have reviewed various research or work done in this area. In^22^, the author proposed a solution for SQLi attack detection based on a Fragmented Query parse tree. Herman^21^ proposed Vulnerability detection using KNN and the Naïve Bayes method, but the attack detection scenario was not considered in this proposed work. In other research like^23–25^ authors have provided solutions for SQLi attack detection using fuzzy technique, LSTM and using generic decryption but these solutions not useful for XSS attack detection. In^26,27^ authors have proposed various solution and models for SQLi attack detection, but these models or solution also does not address the problem of XSS attack. In work done^28^ provided a technique for detection of code injection done via malicious code injection in HTTP request. In^29–31^ researchers have proposed solutions for the detection of XSS attacks, but SQLi attack problems are not considered. So, these are the significant challenges, and there is a need to develop a detection approach that can detect XSS and SQLi attacks and provide high accuracy and low false positive rates. In contrast to the conventional utilization of convolutional neural networks (CNN) and long short-term memory (LSTM) models, our approach involves the development of customized feature extraction methodologies using decoding and standardization of SQL-XSS payload and tokenization and standardization of HTTP requests specifically geared to address the unique attributes associated with SQL injection and cross-site scripting (XSS) attacks. The characteristics above encapsulate the distinct patterns and structures correlated with these hazards, rendering our methodology specialized and exceptionally efficient within this field.

In the following, the contribution of this research can be summarized:

- Developed an approach for the detection of SQL as well as XSS attacks by decoding and standardizing SQL/XSS Payloads or tokenizing HTTP requests.
- We have utilized the CNN and LSTM models in our proposed feature extraction and training approach.
- For this research, we have created our testbed dataset of normal and malicious HTTP requests using the Burp Suite tool extension Burp Logger.
- We have tested our proposed model with Benchmark dataset HTTP CSIC 2010, Sql/XSS Payloads and our testbed dataset and achieved the best accuracy and low false positive rates.
- Our model can detect all types of SQL injection and XSS attacks.

The paper organized a Review of relevant studies described in “Review of relevant studies”. The details of the dataset used in the experimental analysis are described in “Dataset”. Our proposed system description is presented in “Proposed approach”. Evaluation study results and performance comparison are shown in “Evaluation metrics and analysis of result”. Conclusion and future scope of work given in “Conclusions”.

## Review of relevant studies

Several methods can be used to identify SQL injection attacks, including regular expression matching and ML-based models like Support Vector Machine, Naive Bayes, Random Forest, and Decision Tree. Regular expression matching is widely used among these methods due to its high accuracy rate and fast recognition speed. SQL injection attacks have been a consistent focus of network security research and are ranked as the top risk to network applications by the Open Web Application Security Project (OWASP)^15^. Various methods have been proposed to detect SQL injection in recent years by examining the threats, attack types and modes of attack. Standardizing SQL query statements, which unifies query parameter values, SQL keywords, and symbols, has been utilized as a common preprocessing technique.

One downside of this method is that it cannot be applied directly to URLs. SQL injection statements in production environments are complex and varied, making it difficult to standardize them directly. Preprocessing data through the statistics of every word frequency based on a particular method of query sentence segmentation can result in a loss of sentence information and affect the performance of subsequent classification. Counting special characters, words, and statements is a more practical approach to selecting features. Combined with traditional machine learning methods such as Naive Bayes, Random Forest, Decision Tree, Gradient Boosting and Support Vector Machine^22^, it can be used for classification and recognition. Other methods such as regular expression, user behavior and expectation criteria, Hidden Markov Model (HMM)^19^ and database table expansion are also used for detecting SQL injection attacks, but all have limitations. Another method that has been used to detect SQL injection attacks is using a Convolutional Neural Network. CNN imitates the way that living things process visual information. It has been applied to computer vision and natural language processing and may be used for supervised and unsupervised learning. In this context, CNN has also been applied for detecting SQL injection attacks in web applications and database servers by analyzing massive web server and database logs and web HTTP requests. The results of this approach have shown to have high accuracy and effectiveness. One advantage of using CNN for SQL injection detection is that unlike image recognition, where a face can be recognized even if features are misaligned, SQL injection can happen anywhere via URL string or Malicious SQL request to the database server, avoiding the drawback of CNN being susceptible to misaligned features. Additionally, this method effectively retains data information through preprocessing methods and improved pooling layers.

Nofal et al.^23^ proposed a Fuzzy C-Means and Adaptive Neuro-Fuzzy Inference System method for preventing and detecting SQL-based injection attacks. The authors used a testbed dataset and achieved an accuracy of 98.4%. Li Q et al.^24^ used long short-term memory (LSTM) networks for detecting SQLi attacks on intelligent transportation systems. Their research showed an accuracy of 91.53% by using the generation of SQL sample methods and behavior analysis of communication. The method developed by Abaimov and Bianchi^28^ uses a convolutional neural network (CNN) for XSS and SQLi attacks. The authors trained the CNN model on a dataset of XSS payloads, which was taken from GitHub. The study results showed that the proposed method had 95.7% accuracy for SQL injection detection and 90.2% for XSS detection. A novel generic decryption method for SQL queries proposed by Archana Devi et al.^25^ uses a manual decryption method that achieves reasonable accuracy but requires manual human intervention.

Durai et al.^26^ present a novel approach for preventing and detecting SQL Injection attacks using an ontology-based vulnerabilities model. The authors used datasets from the OWASP organization of Open Web Application Security Project and the Database of National Vulnerability to train their model and achieved an accuracy of 92.3% for Cross-Site Scripting (XSS) and 91.05% for SQL Injection (SQLI). However, it is noteworthy that the model is not automated. Another method proposed by Archana Devi et al.^27^ which used query tokenization. The method achieved good results in injecting additional queries and preventing bypass authentication, but it failed to prevent second-order SQL injection, injected union and all union queries and injected alias query. Luo, A. et al.^32^ use CNNs to automatically extract features from the input dataset and train a classifier to detect SQL injection attacks. The authors evaluate the proposed method using three datasets: KDD99, UNSW-NB15, and HTTP CSIC 2010. The results show that the proposed method achieved an accuracy of 98.5% in detecting SQL injection attacks. However, it is only able to detect query-based SQL injection attacks.

Krishnan et al.^29^ presented a DL-based method for cross-site scripting (XSS) attacks using convolutional neural networks (CNNs). They demonstrate the effectiveness of their approach on a dataset of XSS payloads taken from the GitHub repository and achieve an accuracy of 99.59%. DeepWAF is a prototype implemented by Alaoui^33^ for detecting web-based attacks using DL models. This research used LSTM and Word2vec embedding on HTTP CSIC and gained 95.2% accuracy. The novel method was developed by Liu, Z, et al.^30^ for detecting XSS attacks b The approach includes techniques like residual network and GCN for extracting the features of XSS payloads. The experimental results showed a high accuracy of 99.6%. However, the approach is unsuitable for webpages with JavaScript and HTML code, and it takes too much time and effort to train the word vectors. Additionally, the research did not cover real-time detection. Zhang et al.^34^presented a multi-hidden deep neural network and claimed to achieve an accuracy of 96% for attack detection and resolving the overfitting issue. Hackett et al.^31^ used a based neural network for attack detection on three datasets, namely, HTTP CSIC, FWAF and HttpParams datasets and achieved 99.9%.

The approach proposed by Lodha et al.^35^ used the BERT model with a dataset containing 41,770 payloads and achieved 99.9% accuracy. For the detection of XSS and SQLi attacks, Dawadi BR et al.^36^ presented a WAF based on LSTM. In this research, the accuracy for detection of DDoS and SQLi/XSS attacks achieved 97.57% and 89.34%, respectively. In Table 1, we have summarized related studies.

**Table 1:** Comparative summary of relevant study.

Author	Methods	Dataset	Accuracy	Remarks
Deva Priyaa et al.^22^	SVM	Own testbed	95.67	Benchmark dataset not used. Accuracy can further be improved with DL methods
Nofal et al.^23^	ANFIS-FCM	Testbed	98.8	Only testbed data was used. This method can be used only for SQLi attack detection
Li et al.^24^	LSTM	Testbed	91.53	No benchmark dataset was used Detects only SQLi attacks
Abaimov and Bianchi^28^	CNN	GitHub payload data	95.7 SQLi 90.2 XSS	Accuracy can be improved
Durai et al.^26^	Ontology-based rule generation	OWASP, NVD	91.05 SQLi 92.3 XSS	Accuracy can further be improved with DL methods
Luo et al.^32^	CNN	KDD99, UNSW-NB15 HTTP CSIC 2010	98.5	The method only detects SQLi attacks
Krishnan et al.^29^	Ensemble Learning	GitHub repository	99.59	Extract JavaScript keywords can not be used for SQLi attack detection
Alaoui^33^	LSTM Word2vec	HTTP CSIC 2010	95.2	Query standardization not done. Blind SQLi attack not detected in this method
Liu et al.^30^	Graph CNN GCN	XSS payload dataset	99.6	not suitable for webpages with JavaScript and HTML code
Zhang et al.^34^	DNN	Kaggle XSS dataset	96	The method only detects XSS attacks
Hackett et al.^31^	BERT	HTTP CSIC, FWAF and HttpParams	99.9	Stored XSS and Blind SQLi can not be detected in this method
Lodha et. al.^35^	BERT	SQLi payload dataset	99.9	Detects only SQLi attacks
Dawadi et al.^36^	LSTM	ISCX, CISC, and CICDDoS	97.57 DDoS 89.34 SQL/XSS	Other DL methods can lead to some performance enhancement

## Dataset

Our approach for SQL Injection and XSS detection using a CNN LSTM hybrid model utilizes the strengths of both CNN and LSTM models to detect and prevent these types of web attacks effectively. The approach starts by preprocessing the input data, which includes decoding, tokenization, and generalization techniques. The preprocessed data is then fed into the CNN model for feature extraction, and the extracted features are used for training the LSTM model for detection. The LSTM model is trained using a dataset of SQL Injection and XSS payloads, which is collected from different sources such as the OWASP and National Vulnerability Database, and we have also created a dataset using Burp Suite and DVWA. We have also used a payload dataset by combining SQL and XSS payloads^37^ and benchmark datasets, namely HTTP CSIC2010^38^.

### HTTP CSIC 2010

**Figure 2:**
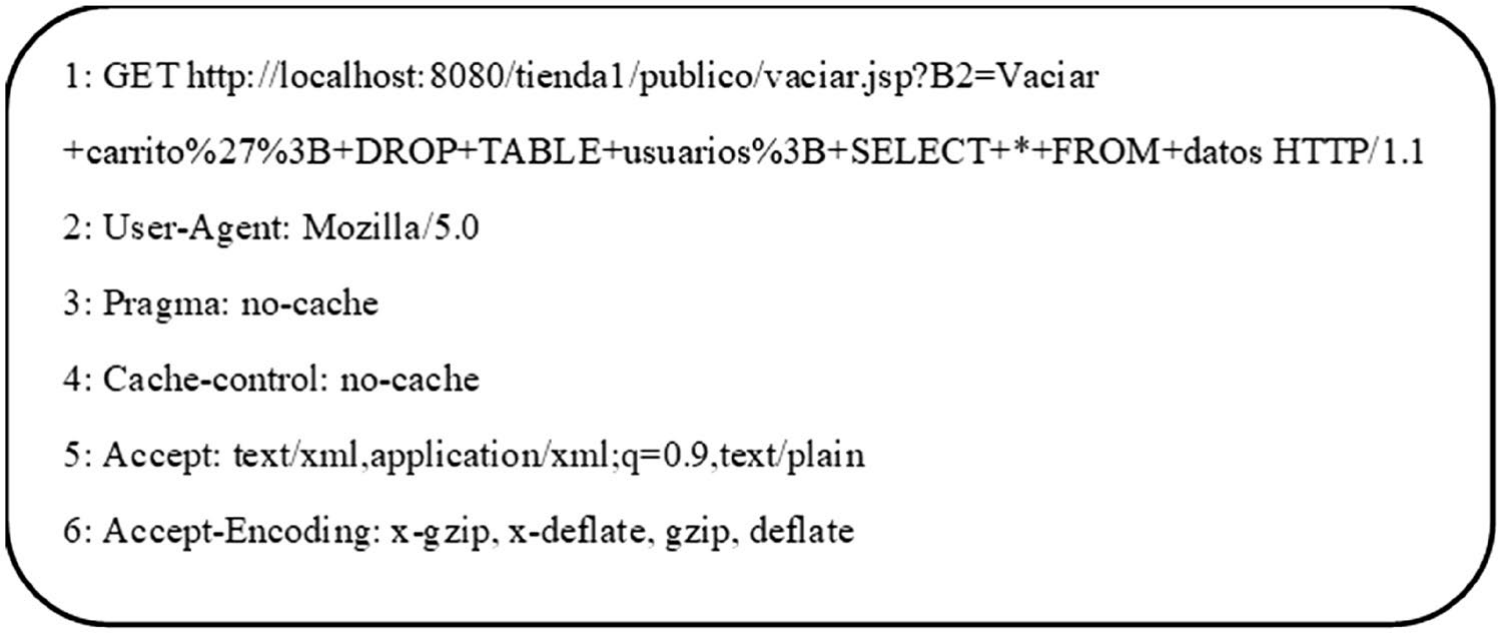
Sample HTTP GET request of malicious SQL query of HTTP CSIC 2010 dataset.

This dataset^38^ was developed at CSIC, containing thousands of automatic web-based requests for normal and attack queries. The traffic was generated by sending regular and malicious requests on an e-commerce web application. This dataset considers three types of malicious requests: static, dynamic and Unintentional illegal requests. Tools like W3AF and Paros have been used to generate attack requests. Buffer overflow, SQL Injection, CRLF Injection, XSS, Parameter tampering, server-side and information-gathering types of attack requests are included in this dataset. It comprises more than 61,000 requests, out of which 36,000 requests were standard requests and other malicious requests. As depicted in Fig. 2, an HTTP request consists of a request line, headers, HTTP-Method, Version of HTTP, Host address and encoding used.

### SQLI–XSS payload dataset

**Figure 3:**
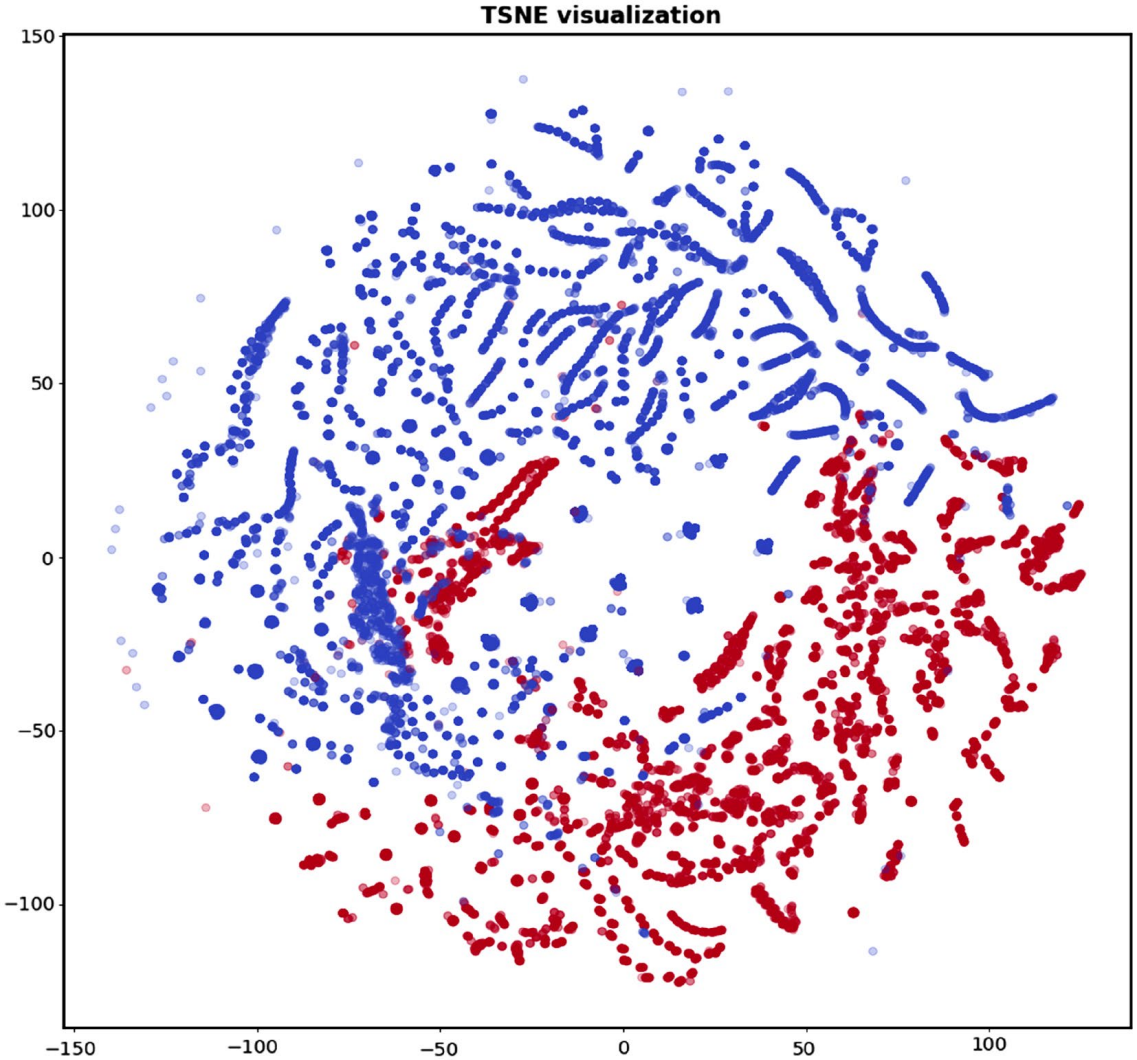
t-SNE visualization of SQLi–XSS payload dataset.

This dataset^39^ is publicly available on the Kaggle for research. It contains various payloads of normal SQL queries and attack-based SQL queries. It contains over 1,00,000 queries labelled 0 and 1 for normal and attack-based payloads. For creating a dataset, we have also used the XSS payload dataset^39^ from Git Hub, which contains around 13,000 normal and attack payloads of XSS attack. In Fig. 3, we can see the t-SNE visualization of features of SQL injection payloads.

### Testbed dataset

**Figure 4:**
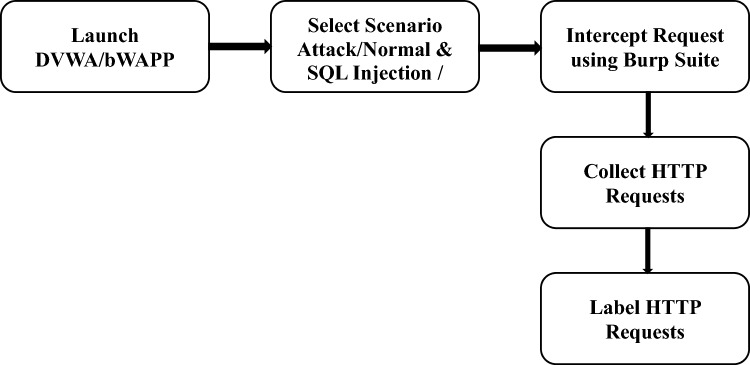
Step-wise process of testbed dataset creation of SQLi/XSS request.

As indicated in Fig. 4, for the creation of the testbed dataset, first, we need to launch Damn Vulnerable Web Application and the buggy Web Application. DVWA and bWAPP are PHP projects with multiple vulnerabilities and are available for testing and research. In the next step, we used Firefox with local proxy and generated requests for all kinds of SQLi and XSS attacks. We have also generated normal HTTP requests. These traffic requests are intercepted using Burp Suite and collected all requests using the Burp Logger extension. Finally, we labelled the HTTP requests as "attack" or "normal".

Using this approach, 11,350 HTTP requests were collected, including SQL injection and XSS attack types. Of the 11,350 requests, 4537 were labelled normal, and the remaining were identified as attacks.

This Testbed dataset has explicitly been curated to evaluate the efficacy of our technique. It serves as a dependable baseline for assessing the success of our methodology.

Table 2 presents the feature list of our testbed dataset. Two characteristics, namely time stamp and Tool, have been removed due to their ubiquity and perceived lack of significance.

**Table 2:** Features included of our testbed dataset.

Feature	Description
Method	The HTTP method used in the request (GET/POST etc.)
Path	The path part of the request's URL, which indicates the resource endpoint
Query	The query string part of the URL includes additional parameters
Paramcount	The number of parameters included in the request
Status	The HTTP status code is returned in the response
Length	The length of the response content in bytes indicates the size of the response data

## Proposed approach

**Figure 5:**
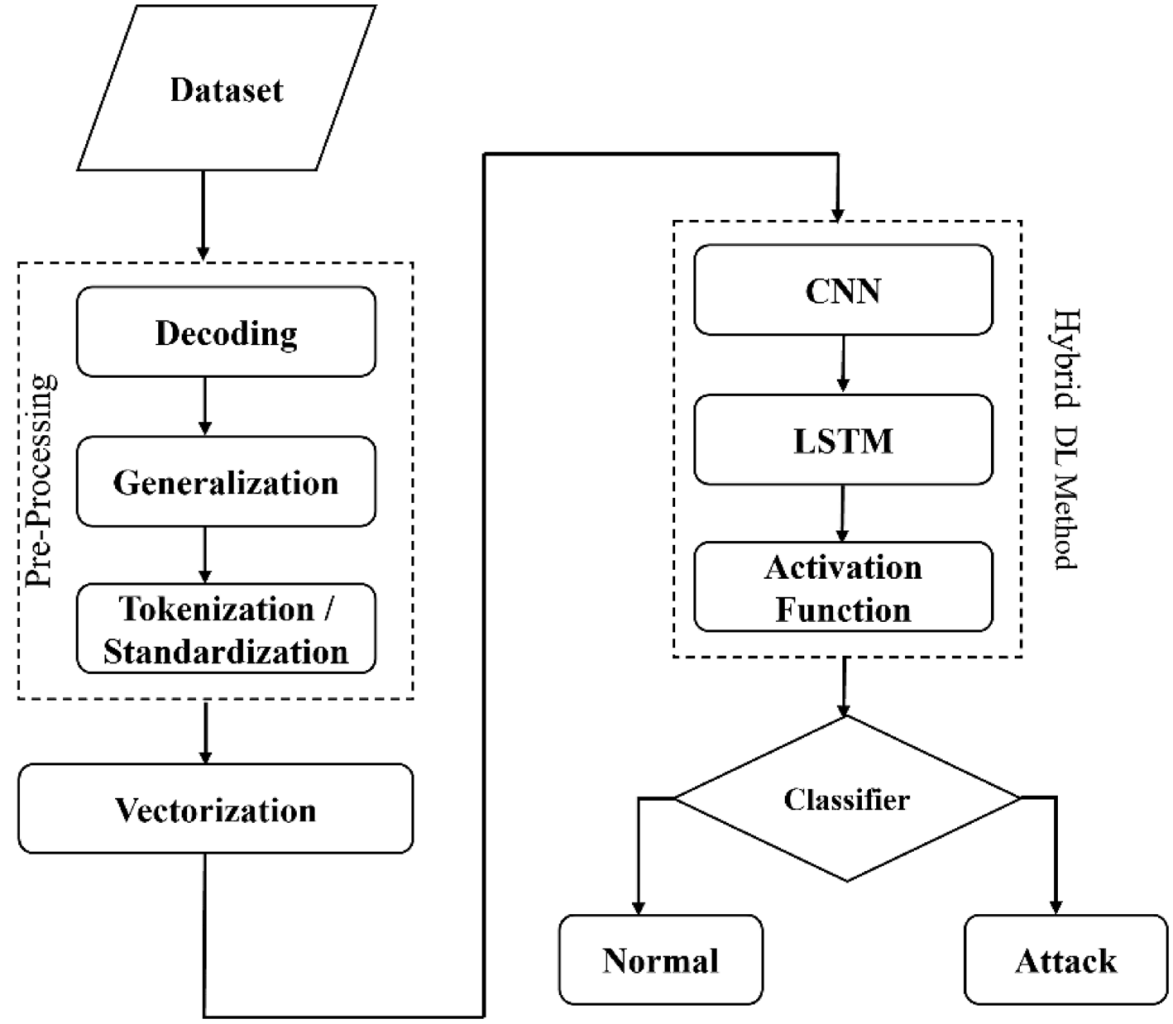
Proposed model for the attack detection.

This section provides a detailed summary of our proposed system developed using deep neural networks like CNN and LSTM approach. The proposed method for detecting XSS attacks and SQL Injection attacks using deep learning primarily utilizes text classification techniques. The input data is first preprocessed through decoding, generalization, query standardization and tokenization. The word2vec model is then used to extract features from the input data. These features are then fed into a CNN-LSTM model to train and classify XSS and standard samples. The architecture of the proposed method is illustrated in Fig. 5. The following subsection can provide further details on the processing steps involved in this method.

### Decoding

Attackers may use encoding techniques like URL encoding, HTML entity encoding, Hex encoding, Unicode encoding, etc., to avoid using regular expressions for traditional filtration or validation. As a result, in this study, we used a decoder for evaluating repeatedly and returning all input data encoding alternatives to their original form. In this phase, hyperlinks are simplified using numbers.

**Figure 6:**
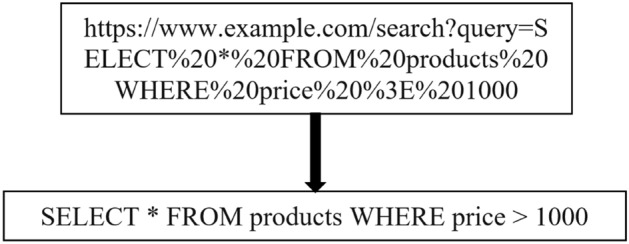
Example of Decoding of HTTP request URL with SQL query.

Once the decoded data has been generalized, the following steps are taken to lessen the disturbance of irrelevant and redundant information: Firstly, we used 'https:/website' to replace several of the input data's URLs. Then, "0" is used instead of the data's numbers. The "param string" is added as a functional input instead of the original string. Additionally, extra unique qualities were removed, including control and blank characters. In Fig. 6, we have shown the process of decoding.

### Tokenization/standardization

**Figure 7:**
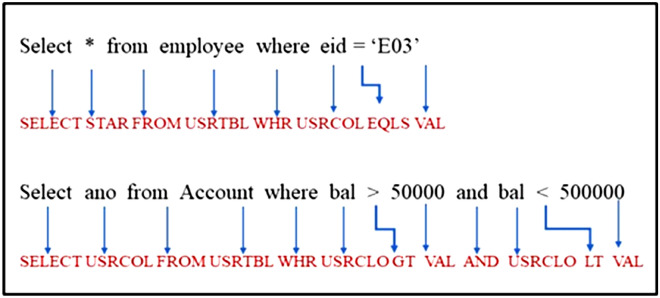
Standardization process of SQL query.

In the next step, tokenization is applied for input data based on features of different scripting languages. Tokenization is used as preprocessing for the XSS attack detection in which we identify starting and ending labels and Windows event and function names, and then unique tokens can be assigned. Each token checked in the vocabulary list. If the token is found, it will be considered else; it will be replaced with a predefined delimiter. For SQL queries, we have used the standardization technique discussed above for the standardization of each element of SQL queries. There are numerous methods for standardization, one of which is depicted in Fig. 7.

### Vectorization

After tokenization and standardization, we have used word2vec for word embedding. For word2vec, the first primary step is vocabulary creation, which consists of the most used common words from the data with tokenization done. Then, in word2vec, we utilized a neural network for calculating the probability of a word appearing based on its context with the help of neighboring words. In the next phase, vector embedding is done using the neural network, which maps with vocabulary.

### Deep learning model

In this part, we have used two deep learning models, CNN and LSTM, which are described in detail.

#### Convolutional neural network (CNN)

The convolutional layer performs convolutions utilizing multiple kernels to extract features from the input data. Each kernel contains trainable weight coefficients, and a bias term can be included to give the network more adaptability. A set of feature maps from the convolutional layer's output is sent to additional neural network layers for additional processing.

This layer serves as the fundamental component of the CNN. The primary responsibility of carrying the network's computing workload is assigned to it. The function of this layer involves the computation of a dot product between two matrices. One of the matrices is referred to as a kernel, which consists of learnable parameters. The other matrix represents the limited section of the receptive field.

Here, ReLU is used as an activation function in convolution operation. ReLU is used due to two main qualities: prevention of gradient disappearance and increasing the speed of the training process. The equation of this function is shown below in Eq. (1).

1 $$f(x)=max(0,x)$$

The function exhibits a behavior where it outputs a value of 0 when provided with any negative input, whereas for any positive input value x, it returns the same value x as the output. Consequently, the output of the system exhibits a continuous range extending from zero to infinity.

After ReLU function in pooling layer max pool function is used. Through down sampling duplicate data, the pooling layer in a CNN seeks to identify invariances and streamline the network. It is accomplished using two basic strategies: maximum pooling, which chooses the pooling outcome to be the maximum value, and average pooling, which chooses the pooling outcome to be the average value.

By reducing the spatial dimensions of the feature maps, these pooling processes increase network efficiency. The neurons inside the fully connected layer exhibit complete connection with all neurons in both the preceding and subsequent layers, similar to what is observed in a standard fully connected neural network (FCNN). Hence, the computation can be performed conventionally using a matrix multiplication, subsequently incorporating a bias factor. The fully connected (FC) layer facilitates the process of establishing a correspondence between the input and output representations.

2 $${y}_{i}=CNN({x}_{i})$$

In above Eq. (2), the input vector ${x}_{i}$ represents the initial input to the CNN network, along with its corresponding class label. The variable ${y}_{i}$ represents the output of the CNN that is intended to be sent as input to the subsequent Long Short-Term Memory (LSTM) network. The feature vector, denoted as ${x}_{i}$, is obtained through the application of the max-pooling operation within a Convolutional Neural Network. The LSTM is provided with input in order to acquire knowledge of long-term temporal relationships.

**Figure 8:**
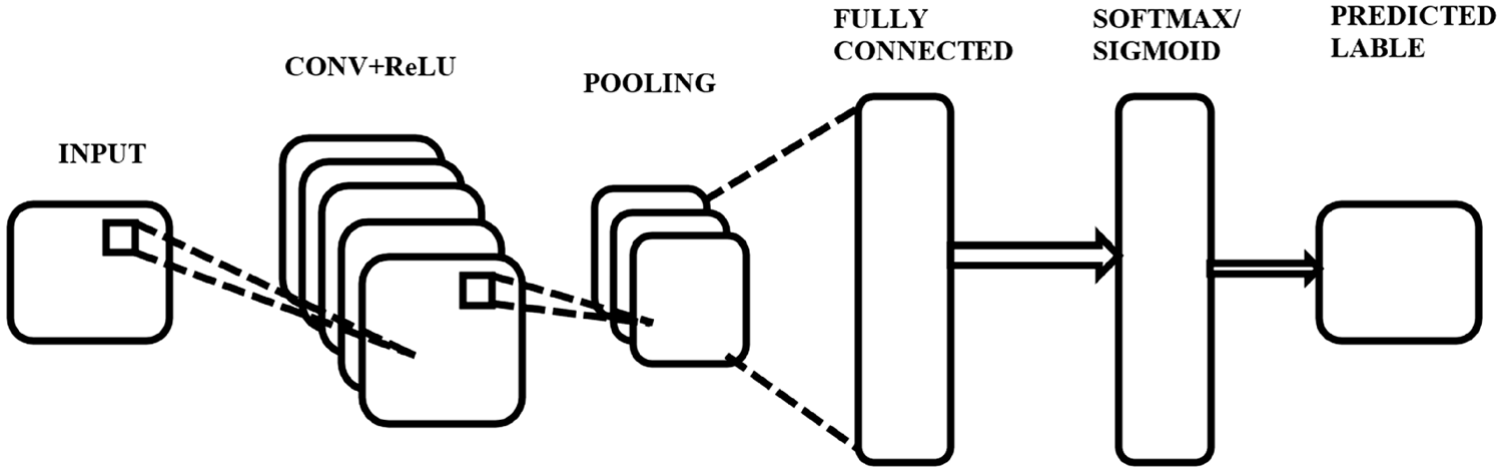
CNN architecture.

In Fig. 8, we can see that the CNN model is made up of a Convolution layer, and the ReLU function is used after that, the Pooling layer is shown in which the Maxpool function is used and at last, the fully connected layer is shown, and after that, we can use sigmoid function or softmax function as per required output label class.

#### LSTM

To overcome the problems of vanishing and exploding gradients, LSTM (Long Short-Term Memory), an improved variation of the Recurrent Neural Network (RNN) architecture, was developed. Traditional RNNs can not handle long-term dependencies as well as LSTMs can, outperforming them in this area. Because of this, previous knowledge can be connected to and retained by LSTMs even when it greatly lags behind the present.

A memory block comprises one or more memory cells and acts as a sophisticated processing unit in an LSTM. These memory cells are essential for aiding in the storage and retrieval of knowledge. The input and output gates are two multiplicative gates included in the memory block. These gates are crucial in regulating all processes inside the memory block. The input gate controls the selective acceptance or rejection of the input flow of memory cell activation. It controls how much fresh data is incorporated into the memory cell. On the other hand, the output gate controls the decision-making procedure regarding transmitting or discarding the memory cell's output state to other nodes. This gate is significant in determining whether the memory cell output is shared with the following layers or used as the LSTM's final output. Compared to conventional RNNs, LSTMs are better at addressing long-term dependencies because they may choose to include or ignore information using these adaptive multiplicative gates. This functionality is beneficial for activities where gathering and using data from far-flung time steps in a series is necessary.

The operation of the LSTM is as follows. The initial stage of our Long Short-Term Memory model involves determining the specific information that will be discarded from the cell state. The determination of this outcome is carried out by a specific layer known as the "forget gate layer," which exhibits sigmoidal behavior. The model examines the values of the previous hidden state and the current input and generates a scalar value ranging from 0 to 1 for each element in the previous cell state. The value of 1 signifies the entire preservation of the item, whereas the value of 0 signifies its complete elimination. The following step is to settle what fresh data will be added to the cell state. Two sections make up this. At the outset, a "input gate layer" constructed from sigmoid nodes chooses which values will be modified. Then, a *tanh* layer generates a vector of potential new state values of the candidate cell state. In the next phase, we'll merge these two to generate an update to the state. In next step to update the previous cell state into the current cell state. we perform the following operations. Firstly, we multiply the old state by the forget gate which allows us to discard information that was deemed irrelevant. Subsequently, we add the product of the input gate, it, and the new candidate values of candidate cell state. These candidate values are scaled by the extent to which we have opted to update each state value. In the final stage of obtaining the output Initially, a sigmoid layer is executed to determine the specific components of the cell state that will be generated as output. Subsequently, the cell state is subjected to the hyperbolic tangent function in order to confine the values within the range of -1 to 1. This transformed cell state is then multiplied by the output of the sigmoid gate, resulting in the selective output of the predetermined components.

**Figure 9:**
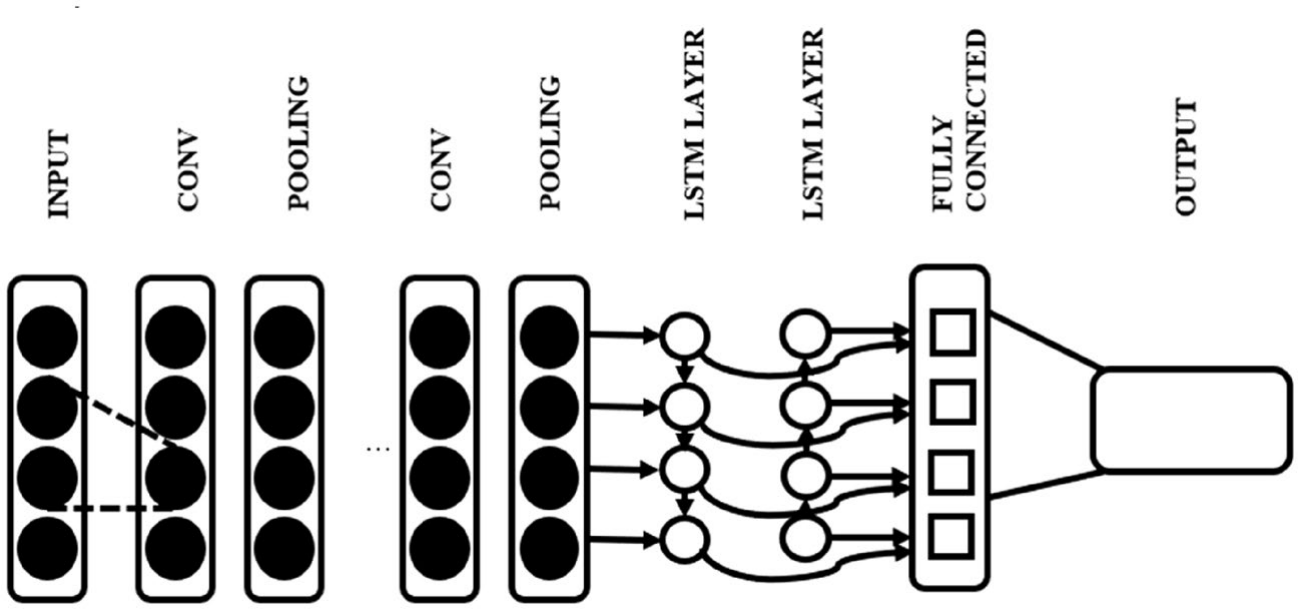
Proposed hybrid model architecture with CNN and LSTM.

Figure 9 shows our proposed model architecture, in which we have used CNN for feature extraction followed by LSTM layers.

The Structure of the hybrid DL model is shown in Table 3. In Table 4, we have shown the hyperparameter setting used for the proposed model. Table 5 displays the environment setup in which experiments have been performed and which libraries and modules have been used.

**Table 3:** Structure of proposed model with parameters and output shape of each layer.

Layer (type)	Output shape	Parameters	Filters	Pool-size
Input layer	[(None, 1000)]	0		
Embedding	(None, 1000, 70)	4900		
Conv1D	(None, 1000, 32)	6752	32	
MaxPooling1D	(None, 500, 32)	0		2
Conv1D	(None, 500, 64)	6208	64	
MaxPooling1D	(None, 250, 64)	0		2
Conv1D	(None, 250, 128)	24,704	128	
MaxPooling1D	(None, 125, 128)	0		2
Flatten	(None, 16,000)	0		
LSTM	(None, 64)	34,560		
Dense	(None, 512)	8,225,280		
Dropout	(None, 512)	0		
Dense	(None, 64)	32,832		
Dense	(None, 1)	65

**Table 4:** Parameter settings of the proposed model.

Hyper Parameter	Value
Convolution filters	128
The kernel size of the filter	4
Fully connected layer	64
Activation function	ReLU
Classification function	Sigmoid
Optimizer	Adam
Epochs	200/50

**Table 5:** Experiment environment setup.

Hardware/software	Version/environment
Operating system	POSIX
CPU	Intel Xeon Gold 6145
Platform architecture	64bit ELF
Memory	96 GB
ROM	16 TB
Graphic card	16 GB NVIDIA QUADRO RTX 5000
Development environment	Jupyter Notebook V3.6
Matplotlib	Version 3.2.0
NumPy	Version 3.1.1
Pandas	Version 1.01
Scikit-learn	Version 0.22.1
Keras	Version 2.13.1
TensorFlow	Version 2.13

## Evaluation metrics and analysis of result

In this section, we have listed evaluation metrics used during the study and then discussed the result analysis.

### Evaluation metrics used in the study

Any machine learning or deep learning model's performance can be evaluated using evaluation metrics, a crucial step in model-building. The type of problem being solved (such as classification or regression) and the objectives of the specific application determine the assessment metric to be used.

Here are some typical evaluation metrics:

Accuracy: this widely used indicator determines the percentage of accurate predictions the model makes. It's outlined as Eq. (3):

3 $${\text{Accuracy = }}\frac{TP + TN}{{TP + TN + FN + FP}}$$

Precision (sensitivity): it is the true positive rate of prediction, which is the proportion of accurately recognized real positives as shown in Eq. (4).

4 $$PRECISION = \frac{TP}{{TP + FP}}$$

Recall (specificity): the proportion of positive examples accurately expected to be positive is measured by recall. Defining recall as shown in Eq. (5).

5 $$RECALL = \frac{TP}{{TP + FN}}$$

F1 score: it is defined as a harmonic-based mean of recall and precision. Defining F1 as shown in Eq. (6).

6 $$F1 = 2 \times \frac{{\Pr ecision \times {\text{Re}} call}}{{\Pr ecision + {\text{Re}} call}}$$

### Analysis and discussion of result

This section thoroughly analyses the outcomes derived from our proposed methodology within the framework of three separate datasets: the Own Testbed dataset, SQL/XSS Payload dataset, and HTTP CSIC 2010 dataset. The datasets above were utilized to assess the efficacy of our methodology in comparison to two widely recognized deep learning methodologies, namely Convolutional Neural Networks (CNN) and Long Short-Term Memory (LSTM) networks. The assessment measures employed in this study encompass Precision, Recall, and F1-Score. These metrics collectively assess our approach's accuracy, comprehensiveness, and balance in identifying SQL injection and XSS attacks.

**Figure 10:**
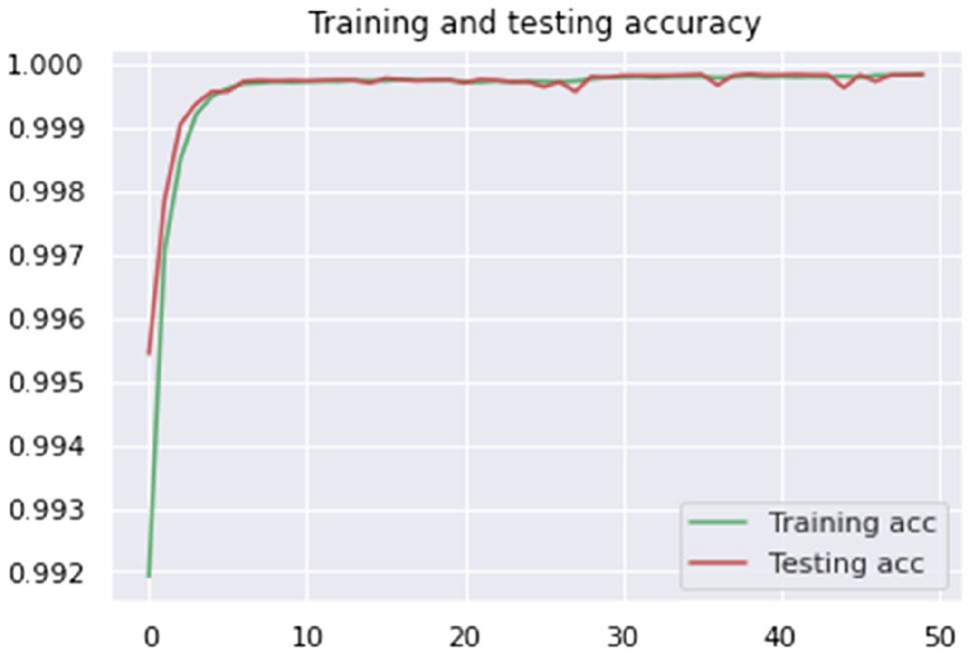
Proposed model accuracy (CSIC 2010 dataset).

**Figure 11:**
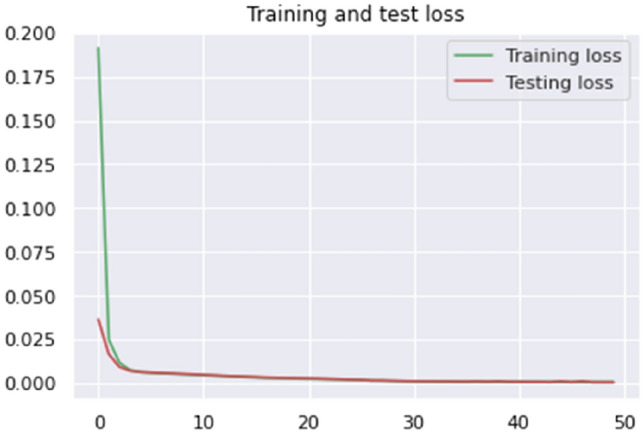
Proposed model loss (CSIC 2010 dataset).

Figures 10 and 11 show our HTTP CSIC 2010 fraudulent request detection success. After 50 epochs of training, our model achieves 99.77% accuracy. This astonishing result shows the model's ability to identify legitimate and fraudulent dataset requests. The convergence of our model and the decrease of the loss function to 0.0001 demonstrate its ability to capture web application assault patterns and features.

**Figure 12:**
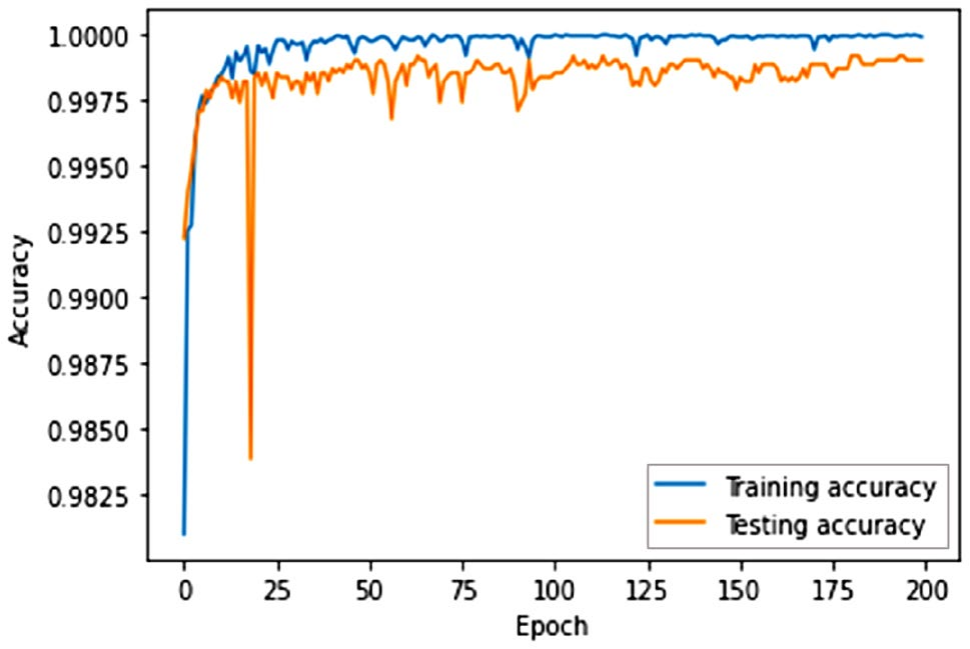
Proposed model accuracy (SQLi–XSS payload dataset).

**Figure 13:**
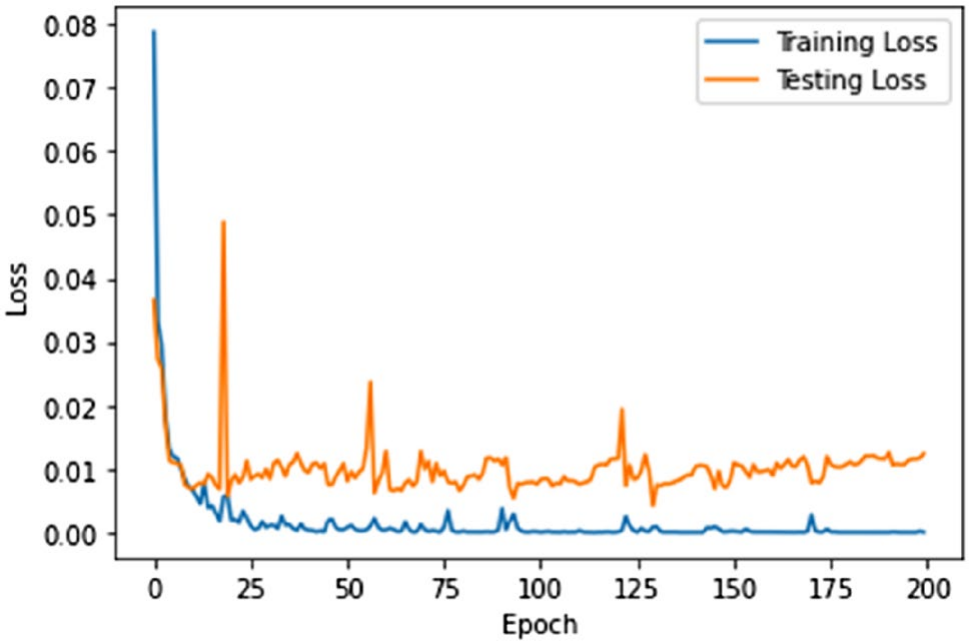
Proposed model loss (SQLi–XSS payload dataset).

The accuracy of our model on the SQLi–XSS Payload dataset was 99.84%. It demonstrates the efficacy of the model in identifying and detecting harmful payloads. The accomplishment above is demonstrated by the decrease in losses to 0.01, as depicted in Figs. 12 and 13.

**Figure 14:**
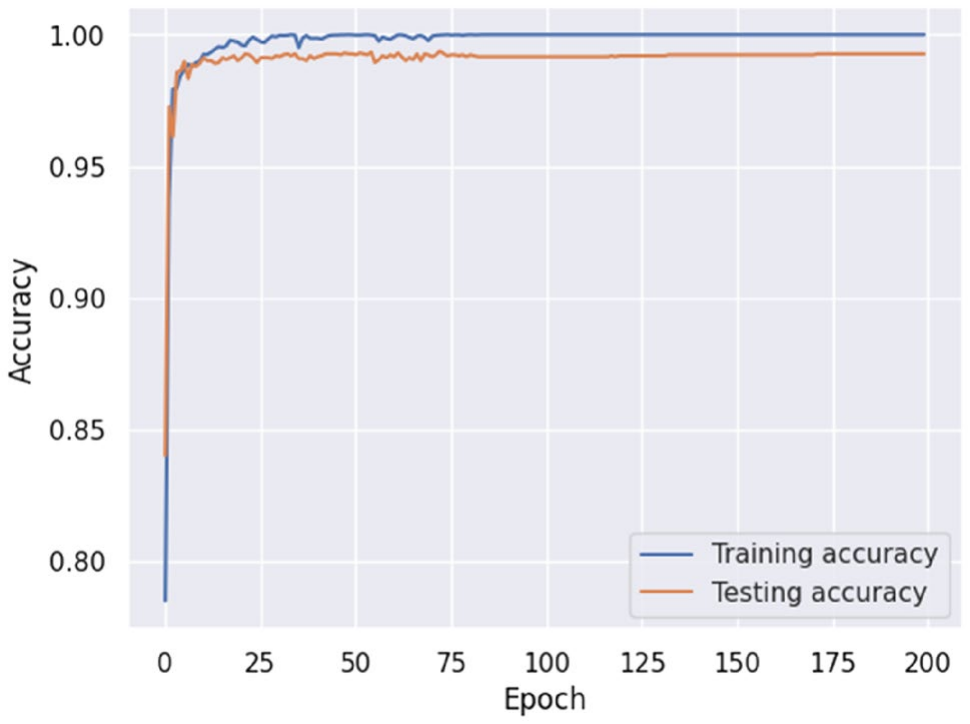
Proposed model accuracy (own testbed dataset).

**Figure 15:**
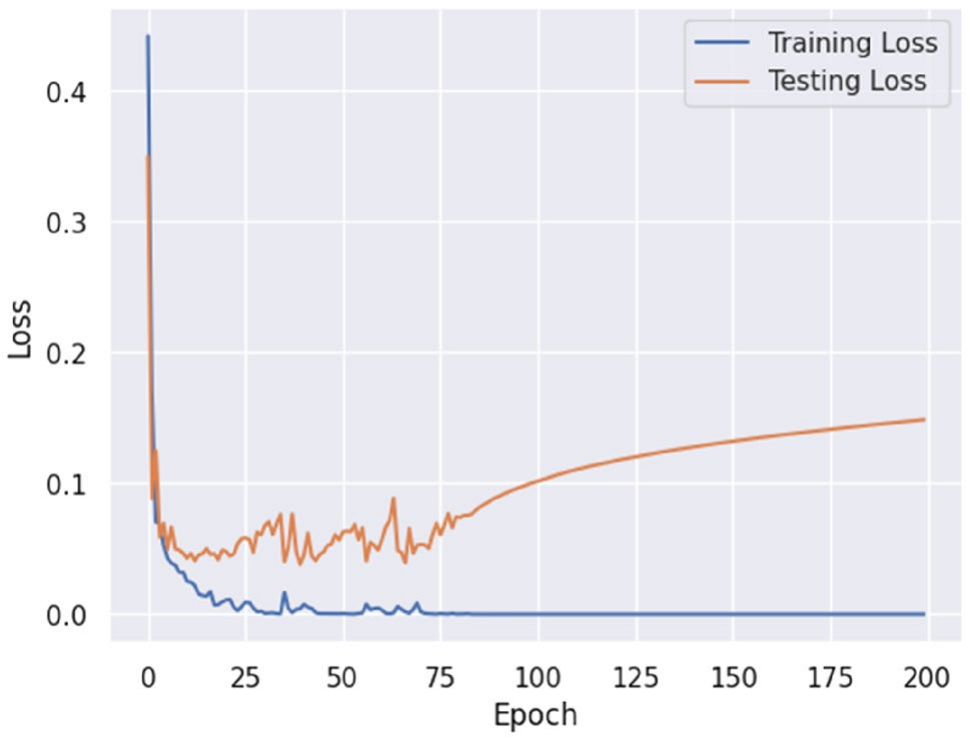
Proposed model loss (own testbed dataset).

Additionally, our model exhibited a 99.23% accuracy rate when tested on the Our Testbed dataset, showcasing its proficiency in classifying diverse attack scenarios. The loss measurements depicted in Figs. 14 and 15 provide insights into the learning capacity of the model.

**Figure 16:**
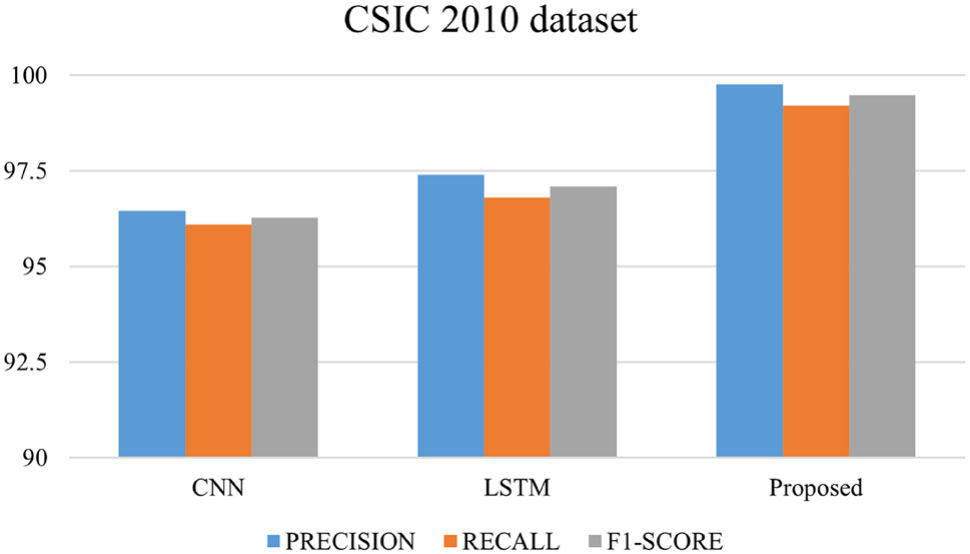
Comparative analysis of metrics: performance evaluation of our proposed model in contrast to CNN and LSTM models on the HTTP CSIC 2010 dataset.

**Figure 17:**
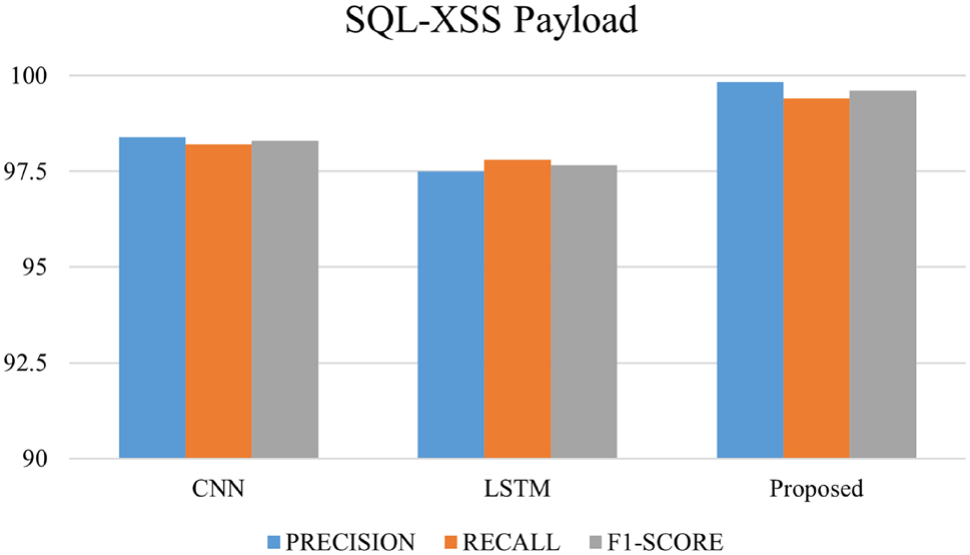
Comparative analysis of metrics: performance evaluation of our proposed model in contrast to CNN and LSTM models on the SQL–XSS payload dataset.

**Figure 18:**
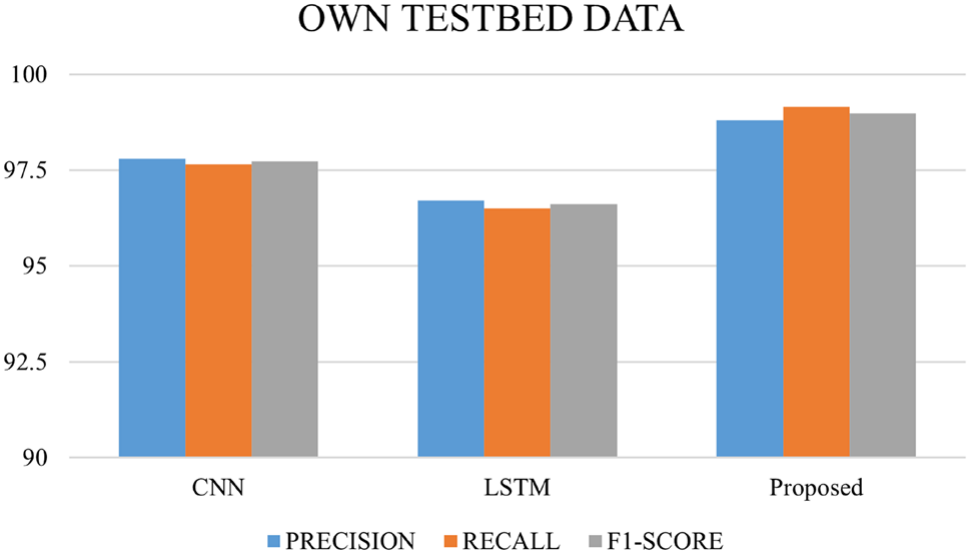
Comparative analysis of metrics: performance evaluation of our proposed model in contrast to CNN and LSTM models on the testbed dataset.

After 75 epochs, we saw overfitting concerns in our testbed dataset across 200 epochs. The Adam optimizer was employed, and epoch wise average time for the Payload and Testbed data was 206.02 s and 88.70 s, respectively. Figures 16, 17, and 18 depict the precision, recall, and F1 score for the three datasets and a comparison with the CNN and LSTM models.

The outcomes of applying the CNN, LSTM and hybrid models to three distinct datasets—own testbed data, SQL–XSS Payload, and HTTP-CSIC 2010 might offer insightful information about how well the models perform on various kinds of data. The proposed method identified assaults with 98.8% precision and low false positives. Our technique had a 99.15% recall rate, indicating its ability to capture most strikes. The method's precision-recall balance was shown by its 98.97% F1-Score. We can see that our proposed model gives good results in terms of precision, recall and F1 Score as compared to CNN and LSTM models in all three datasets. Our model achieved a precision of 99.76, a recall of 99.84, and an F1 Score of 99.72 with the SQLi/XSS Payload dataset. For the HTTP CSIC 2010 dataset, precision has a low false positive rate of 99.76%, protecting real traffic. A 99.21% recall rate suggests a high attack capture rate. The method's 99.48 F1-Score shows its ability to balance precision and recall.

Table 6 summarizes model performance on a dataset. With balanced precision and recall, the "AE-LSTM" model has an F-Score of 81.96 and 87.26% accuracy. The "CNN" model has an F-Score of 99.49 and an accuracy of 99.5% because of its near-perfect precision of 98.98 and lesser recall of 1. With 98.69% accuracy, excellent precision, and recall, the "LSTM" model has a 97.82 F-Score. Meanwhile, the "Proposed" model outperforms all others with 99.84% accuracy, high precision, and recall, earning a 99.82 F-Score. Our model is well-suited to the dataset and job, indicating its potential to improve web application security against the dangers evaluated.

**Table 6:** Performance comparisons.

Model	Accuracy	Precision	Recall	F-score
AE-LSTM^40^	87.26	81.22	89.7	81.96
CNN^41^	99.5	98.98	1	99.49
LSTM^42^	98.69	99.85	95.69	97.82
Neural Network^43^	95	–	–	–
CNN^44^	97.07	–	–	97.51
SQLNN^34^	96.16	97.28	92.23	94.68
Proposed	99.84	99.76	99.88	99.82

## Conclusions

In conclusion, we have identified the problem of detecting all kinds of SQLi and XSS attacks using a single model and securing web applications against XSS and SQL injection (SQLi) attacks has demonstrated promising results when using a hybrid combination of CNN and LSTM approaches. This method effectively detects and categorizes security threats in real time by utilizing the strengths of both the CNN and LSTM models. We have created a testbed dataset using Burp and evaluated our proposed model with three datasets. Our model provided high accuracy and very low false positive rates in all the experiments with all three datasets. Our model has achieved 99.77% accuracy with the HTTP CSIC 2010 dataset, 99.84% accuracy with the SQLi/XSS Payload dataset, and 99.23% with our testbed dataset.

Future studies may look into other architectures and methods for fusing CNN and LSTM models to enhance the precision and resilience of the models. Incorporating additional forms of data, such as network metadata, may offer valuable data for identifying security issues. Extending the models to other security-related activities, such as identifying malicious URLs, phishing attempts, or botnet attacks, is another subject for future research. The hybrid mix of CNN and LSTM models can play a vital role in safeguarding web applications and defending against cyber threats by combining various data types and utilizing cutting-edge machine-learning techniques. With further development and refinement, these models have the potential to become a vital tool for protecting against a wide range of cyber threats. We can use other scaling and normalization methods to reduce the time taken for model training. In future, we can apply this model to another dataset to detect other types of attacks like Zero-day attacks and DDoS attacks.

## Data Availability

The datasets used and analyzed during the current study are available from the corresponding author upon reasonable request.
